# Relatively high light inhibits reserves degradation in the *Coptis chinensis* rhizome during the leaf expansion by changing the source-sink relationship

**DOI:** 10.3389/fpls.2023.1225895

**Published:** 2023-09-01

**Authors:** Wenjia Ke, Yirou Li, Furong Zhong, Maoyao Pen, Jijing Dong, Binjie Xu, Yuntong Ma, Tao Zhou

**Affiliations:** ^1^ State Key Laboratory of Southwestern Chinese Medicine Resources, Chengdu University of Traditional Chinese Medicine, Chengdu, Sichuan, China; ^2^ College of Pharmacy, Chengdu University of Traditional Chinese Medicine, Chengdu, Sichuan, China; ^3^ Innovative Institute of Chinese Medicine and Pharmacy, Chengdu University of Traditional Chinese Medicine, Chengdu, Sichuan, China

**Keywords:** source-sink, leaf, *Coptis chienesis*, light intensity, foliar P

## Abstract

The early spring is a seasonal high-light “window” for new leaf growth and photosynthetic carbon capture by the shade-tolerant evergreen understory plants. However, it remains unclear how light regulates the source–sink relationship between rhizome (RO), mature leaf (ML), and immature leaf (IL) during *Coptis chinensis* leaf expansion. Understanding this relationship is essential to reducing RO reserve degradation and ultimately promote RO biomass accumulation. The plants grew in an artificial climate chamber with low (50 μmol m^−2^ s^−1^) and relatively high (200 μmol m^−2^ s^−1^) light intensity treatments. Leaf fluorescence, foliar phosphorus (P) fractions, soluble sugars, starch, total P, and alkaloid concentrations in ILs, MLs, and RO were measured, and ^13^C labeling was used to indicate the direction of photosynthetic carbon flow between organs. The plants grown under high light intensity had higher levels of starch in RO and higher RO biomass at the end of the year compared to those grown under low light intensity. The photosystem II (PSII) operating efficiency [*Y*(II)], relative electron transport rate (*r*ETR), and photochemical quenching (qP), as well as sucrose and glucose, in ILs and MLs under relatively high light, was higher than those under low light. The glucose and starch concentrations in ILs at 35 d was significantly higher than that at 15 d when plants were under 200 μmol m^−2^ s^−1^, while they were not significantly changed and remained low at 50 μmol m^−2^ s^−1^. The ^13^C was detected in the RO when plants were grown at 200 μmol m^−2^ s^−1^, regardless of ILs and MLs ^13^C labeling, while no ^13^C was detected in the RO when plants were under 50 μmol m^−2^ s^−1^. Additionally, the proportion of photosynthetic transport from ILs to MLs was significantly higher than that from MLs to ILs under the 50-μmol m^−2^ s^−1^ limit. Total P concentration in ILs was lower under relatively high light, but there was no difference in nucleic acid P concentration in ILs under the two light intensity treatments. The alkaloid concentration in RO was lower under 200 μmol m^−2^ s^−1^ than that under 50 μmol m^−2^ s^−1^. We propose that relatively high light reduces the need for carbohydrates and P stored in the RO to support IL growth by (1) accelerating the sink-to-source transition in ILs, which inhibits the use of reserves in the RO; (2) using energy from MLs to support IL growth, thereby reducing RO reserve consumption, and (3) reducing the demand for P by investing less in the development of photosynthetic machinery. Furthermore, under low light, MLs serve as a sink and rely on other organs for support, directly or indirectly exacerbating the reserves lost in the RO.

## Introduction

*Coptis chinensis* is a perennial, shade-tolerant, evergreen, understory medicinal plant. The rhizome (RO) of *C. chinensis*, which contains alkaloids, especially berberine, is the main effective component for its therapeutic effects, such as immune-enhancing, hepatoprotective, treating diabetes, Alzheimer’s disease, etc. (Jiang et al., 2013; Xu et al., 2015; Li et al., 2018). *Coptis* ROs are also widely used in other East Asian countries such as Korea and Japan, with an annual demand of 3,500–4,000 tons, which continues to rise every year. China accounts for two-thirds of the global production. Owing to overharvesting, global warming, and dimming, the wild resources of *C. chinensis* have almost been depleted, and over 90% of the medicinal herb is produced by cultivation (IUCN, 2004; Li et al., 2020). Cultivation practices involve shading *C. chinensis* with shade cloth throughout the growing period (**Supplementary Figure S1**), which takes 6–8 years to produce a harvest, resulting in low yields and high costs. As the demand for *C. chinensis* herbs exceeds supply, improving cultivation technology is crucial.

Understory plants in deciduous forests leaf out earlier in spring than do conspecific canopy trees, although there are some exceptions, such as in *Fagus grandifolia*, *Fagus crenata*, and *Betula lenta* (Tomita and Seiwa, 2004; Richardson and O’Keefe, 2009). The earlier leaf out allows understory plants to take advantage of the relatively high light conditions before canopy closure, which is considered a mechanism that allows them to thrive in the understory despite low summer light availability (Gill et al., 1998; Seiwa, 1999; Augspurger et al., 2005; Kwit et al., 2010). Estimates show that the early-leafing species intercept 33%–98% of their growing season total irradiance before canopy closure (Augspurger et al., 2005). Indeed, the high light intensity window in the spring is critical for the growth and survival of the understory plants (Augspurger, 2008; Lopez et al., 2008; Richardson and O’Keefe, 2009; Osada and Hiura, 2019).

*C. chinensis*, like most understory evergreens in winter deciduous forests, experiences new leaf emergence and expansion in the early spring, before the canopy is closed by the upper canopy trees (Augspurger et al., 2005; Ida and Kudo, 2008; Liao et al., 2021; Liu et al., 2021). High light intensity promotes leaf growth and biomass accumulation in understory plants before canopy closure by mediating leaf physiological and morphological changes that have been well documented (Kwit et al., 2010; Heberling et al., 2019). The leaf development of shade-tolerant understory species, especially delayed-greening species, is divided into three stages: rapid leaf expansion, developing photosynthetic capacity, and maturing as a source organ (Czech et al., 2009). In the first stage, immature leaves (ILs) act as a sink and receive carbohydrates and nutrients from the source organ (e.g., mature leaves, rhizome, and stem) for their own growth (Turgeon, 1989; Vitoria et al., 2016). In the second stage, the transition from being a sink to becoming a source occurs, and the earlier the transition occurs or the greater the photosynthetic capacity of the ILs, the better it is for reducing reserve consumption in the source organ (Turgeon, 1989; Turgeon, 2006). Finally, the leaves act as a source of support for the growth of sink organs (Czech et al., 2009). The rate of transition and photosynthetic capacity of ILs are controlled by multiple environmental signals, especially light (Lake et al., 2001; Lim, 2003; Trouwborst et al., 2011; Fotis and Curtis, 2017). However, it remains unclear how the light intensity regulates the photosynthetic capacity and sink-to-source transition rate of ILs, thereby reducing reserve consumption in the RO of *C. chinensis*. Understanding the underlying mechanisms of the trade-off between reserve storing and supporting new leaf growth regulated by light intensity in the spring is beneficial for better understanding the survival strategies of understory plants and for exploring high-yield cultivation practices in *C. chinensis*.

Light plays a crucial role in plant growth, development, and morphology, while also serving as a signal for altering the relationship between sinks and sources (Hangarter, 1997; Valladares and Niinemets, 2008; Shafiq et al., 2021). This is because light directly influences photosynthesis and the demand for metabolites by sinks, which indirectly regulates the redistribution of reserves (Iqbal et al., 2012). Low light intensity can cause a shortage of photosynthates and nutrients, forcing plants to transport them from storage organs to shoot tissues to meet growth demands (Lynch and Brown, 2001; Myers and Kitajima, 2007; Franklin, 2008; Obendorf et al., 2009). In contrast, excess carbohydrates produced under high light are stored in leaves or transferred to sink organs, indirectly inhibiting their export from the source once reserves are full (Rossi et al., 2015; Zhou et al., 2021). For example, high light increases the photosynthetic capability of saffron seedling leaves, reducing the carbohydrate demand from leaf development in the source organ and leading to reduced carbohydrate consumption in the mother corm (Zhou et al., 2022). Furthermore, in shade-intolerant plants such as corn, soybean, wheat, rice, and *Arabidopsis*, low light intensity speeds up the senescence of mature leaves (MLs), resulting in the transfer of carbohydrates and nutrients to sink organs. On the other hand, low light does not induce senescence in the MLs of shade-tolerant plants, as they adapt by reducing their respiratory consumption to ensure survival (Reich et al., 1998; Yao et al., 2017). These findings indicate that the role of MLs as a source for supporting new leaf growth is environmentally dependent, especially in light conditions.

The concentration of phosphorus (P) in leaves under high light conditions has been found to be lower than that under low light conditions (Cheng et al., 2014; Kuppusamy et al., 2021). However, it remains uncertain whether high light reduces the P requirement during the expansion stage of ILs. During this stage, plants require a higher proportion of P compared to nitrogen and potassium to support rapid growth. This is because organisms must allocate a disproportionately greater amount of P to rRNA to meet the protein synthesis demands of growth (Hughes et al., 2007; Lambers, 2022). Various studies have shown that plants can maintain their photosynthetic capability with lower foliar P concentrations when exposed to high light intensity compared to those in low light, including *Lupinus abnus* L., *Glycine max* (Linn.) Merr., *Zea mays* L., and Proteaceae (Cheng et al., 2014; Hayes et al., 2018; Zhou et al., 2019a; Zhou et al., 2019b; Kuppusamy et al., 2021). These results imply that, at least in part, the P needed for the development of the photosynthetic capacity of ILs under high light may be lower than that required under low light during the transition phase from sink to source, potentially leading to a reduction in P consumption in source organs.

In the main distribution region of *C. chinensis* in southwest China, the total irradiance generally ranges from 3,350 MJ m^−2^ year^−1^ to 4,190 MJ m^−2^ year^−1^, which falls below the annual average of 5,900 MJ m^−2^ year^−1^ for China (Zhou et al., 2021). Given the low radiation levels in this area, the high light experienced during early spring is particularly valuable for promoting the growth of *C. chinensis*. Although, the influence of light intensity on leaf development and its ultimate effect on rhizome biomass, *via the* regulation of reserve flow between source and sink organs, has not been clearly understood. To fill this knowledge gap, we conducted a systematic study of *C. chinensis* under low and relatively high light intensities in a greenhouse setting. The photochemistry efficiency (*F_v_/F_m_*; *Y*(II); qP; *r*ETR; *NPQ*) in ILs and MLs, foliar P concentration and fractions, and carbohydrate contents in leaves and RO were evaluated. To evaluate the direction and intensity of carbon movement among ILs, MLs, and RO, we employed ^13^C isotopic labeling, a well-established method for tracking the pathway of reserves in plants (Vitoria et al., 2016; Zhang et al., 2021). Specifically, during the leaf expansion stage, we formulated the hypothesis that (1) increasing the light intensity appropriately promotes the transition from sink to source in ILs, resulting in a decrease in reserve (carbohydrates and P) consumption in RO; and (2) as the temperature rises in the spring, under low light, MLs do not function as a source and may even act as a sink organ.

## Materials and methods

### Plant materials

The 3-year-old *C. chinensis* used in the present study were collected from the cultivation base in Pengzhou, Sichuan, China (31° 10′ N, 103° 50′ E). The average daily sunshine duration, air temperature, and precipitation for the last 20 years (2000–2020) were obtained from the nearby meteorological station and are shown in **Supplementary Figure S2**. During the period of leaf expansion (early spring), which had the highest number of daily sunshine hours throughout the year, the range of sunshine hours was between 4 h and 5.5 h (**Supplementary Figure S2**). Before the experiment was conducted, the plants were transplanted (February 2022) from the Pengzhou to the Chinese Medicine plant garden of Chengdu University of Traditional Chinese Medicine (30° 68′ N, 103° 81′ E) for 1 month of acclimatization. The plants were grown individually in plastic pots (13 cm^3^ × 15 cm^3^). An ochric aquic cambosol soil was obtained from the surface layer (0–20 cm) of farmland in the plant garden. The soil was air-dried, sieved to 2 mm, and thoroughly mixed, and properties were as follows: pH (1:2.5, soil:water) was 6.31, organic matter content was 24.1 g kg^−1^, total N was 1.6 g kg^−1^, available N was 120 mg kg^−1^, Olsen-P was 15.3 mg kg^−1^, and available K was 110 mg kg^−1^. Each pot was filled with 5 kg of air-dried soil. As the fertility of the soil was adequate for plant growth, no extra mineral fertilizers were added to the soil. All pots were watered daily until they were moved to the greenhouse.

### Experimental design and plant growth conditions

The experiment was a randomized block design in an artificial climate chamber with 85% relative humidity and a day/night temperature of 20°C/10°C. According to the daily sunshine hours during the period of leaf expansion, a photoperiod of 8 h day/16 h night was employed. A light-emitting diode (LED), with wavelengths ranging from 380 nm to 800 nm, provides the irradiance, with a red-to-blue ratio of 3:1. The lamps were evenly distributed on the panel with a 4-cm row distance, a 5-cm lamp distance, and increased density around the panel to ensure uniformity and stability of light intensity under the lamp (**Supplementary Figure S3**). Usually, 0%–4% and 4%–12% of the open-sky light are approximately closed forest understory and tree-fall gap light environments, respectively (Canham et al., 1990; Walters and Reich, 1999). In combination with the light intensity of the growing area (canopy light intensity at noon averages about 2,000 μmol m^−2^ s^−1^) of *C. chinensis* (**Supplementary Figure S4**), two light intensities were employed: 50 μmol m^−2^ s^−1^ ± 5 μmol m^−2^ s^−1^ (low light) and 200 μmol m^−2^ s^−1^ ± 5 μmol m^−2^ s^−1^ (relatively high light), which represent 2.5% and 10% of the canopy light, respectively. Our previous study using a fast light response curve also found that a light intensity of 200 μmol m^−2^ s^−1^ is typical for the cultivation of *C. chinensis in the* laboratory (**Supplementary Tables S1**, **S2**), although it is much lower than natural light intensities found in field conditions. Light intensity was measured using a quantum sensor (UPRtek, PG100N, Taiwan, China).

When the new leaves were ready to appear (March 10, 2022), the uniformly growing and healthy plants in pots were transferred from the Chinese Medicine plant garden to the artificial climate chamber. The arrangement of pots under the LED panel is shown in **Supplementary Figure S3**. The pots were arranged in a diamond shape, with a 20-cm distance between two pots to ensure that the plants were unobstructed from each other and received almost the same amount of light from the LED. A distance of 50 cm was maintained between the canopy and the light to eliminate the effect of temperature produced by the lamp in the two light conditions.

Plants from both light treatments were transplanted back to the Pengzhou base after the controlled experiment, and the field management after transplanting was the same as the local cultivation practice. The plants were dug up in October to determine the dry weight of the RO.

### Plant sampling

The chlorophyll fluorescence parameters of the ILs and MLs were measured at 15 d (ILs fully expanded and pale green; MLs grow normally dark green) and 35 d (ILs fully expanded and dark green; MLs with no obvious changes) after the start of the light treatment, respectively. The ILs of fresh plants were cut for the determination of pigment content. Plants were harvested 15 d and 35 d after the start of light treatment, frozen in liquid nitrogen, and stored at −80°C for subsequent index determination.

### Chlorophyll *a* fluorescence

The chlorophyll fluorescence of ILs and MLs, respectively, was measured using a MINI-PAM-II Portable Fluorometer (Walz, Effeltrich, Germany) *in situ* in complete darkness in an artificial climate chamber on day 15 and day 35 of light treatment. The leaves were dark-adapted for 30 min, and then a 0.6-s saturating light pulse (1,100 μmol m^−2^ s^−1^) and an actinic light for 60 s (261 μmol m^−2^ s^−1^) were applied to obtain the minimal and maximal fluorescence yield (*F*_0_ and *F_m_*). The maximum light-adapted fluorescence (*F_m_’*) and steady-state fluorescence (*F_s_*) were then measured with continuous actinic light. The PSII operating efficiency [*Y*(II)], relative electron transport rate (*r*ETR), photochemical quenching (qP), the maximum photochemical efficiency PSII (*F_v_/F_m_*), and the nonphotochemical dissipation of absorbed energy (NPQ) were calculated with the following formula (Kooten and Snel, 1990; Oquist and Chow, 1992):


(1) $$Y\left(\text{II}\right)=\left(F_{m}^{'}-F\right)/F_{m}^{'}$$

(2) rETR=Y(II)×PAR×0.84×0.5

(3) $$\text{qP}=(F_{m}^{'}-F_{s})/\left(F_{m}^{'}-F_{0}\right)$$

(4) $$F_{v}/F_{m}=(F_{m}-F_{0})/F_{m}^{'}$$

(5) $$\text{NPQ}=(F_{m}-F_{m}^{'})/F_{m}^{'}$$

### Determination of chlorophyll concentration

After 15 d and 35 d of treatment, fresh ILs were cut and crushed immediately with liquid nitrogen, and 0.1 g (fresh weight (FW)) of powder was soaked in 10 mL of 80% v/v acetone (20% ethanol) at 25°C to extract chlorophyll for 24 h in the dark (Lichtenthaler and Buschmann, 2001). Powdering the leaves speeds up the leaching of chlorophyll and shortens the steeping time. The absorbance at 645 nm and 663 nm was measured with an enzyme-labeled instrument (SpectraMax iD3, Molecular Devices, USA), and the chlorophyll concentration was calculated with the following formula (Arnon, 1949; Porra et al., 1989).


(6) Chlorophyll a (Chl a)=(12.7×A663−2.69×A645)×V/(1,000×W)

(7) Chlorophyll b (Chl b)=(22.9×A645−4.68×A663)×V/(1,000×W

Where *V* is the total volume of extract (mL) and *W* is the leaf fresh weight (g).

### Determination of soluble sugar and starch

Soluble sugars from lyophilized (FDU-2110, EYELA, Japan) leaves and RO (dry weight (DW)) were extracted with 80% v/v ethanol at 80°C for 10 min, and the supernatants of the two extractions were combined to prepare soluble sugar samples for analysis. Soluble sugar was measured directly in the extract by a Waters Binary HPLC System (Waters 1525-2707, Milford, USA) equipped with a refractive index detector (2414, Waters, Milford, USA). The analytical conditions were as follows: column Agilent Hi-Plea Ca (8% crosslinked) 300 mm × 7.7 mm, 8 μm in diameter (Agilent Technologies Inc., USA); column temperature 85°C; mobile phase Milli-Q water; flow rate 0.6 mL min^−1^ (Zhou et al., 2022). Data were collected and processed by the Waters Chromatography Station DataApex. Sugars were identified by comparison with retention times and coelution of authentic standard solutions.

The residue after ethanol extraction was washed several times with ultra-pure water and used for starch analysis. The precipitate was gelatinized at 100°C for 10 min, and the process was repeated once. The resulting supernatants were combined and treated with perchlorate before analysis. The starch content was determined by taking an appropriate amount of sample by the anthrone method (Setter and Ella, 1994).

### Observation of chloroplast ultrastructure and starch by transmission electron microscope

Small squares of 1 mm^2^ size were cut from the ILs after 35 d of growth in the light resource and immediately placed in 3% glutaraldehyde for prefixation. The tissue was then postfixed in 1% osmium tetroxide, dehydrated in series acetone, infiltrated in Epox 812 for a longer period, and embedded. The semithin sections were stained with methylene blue, and ultrathin sections (EMUC7, Leica, Germany) were cut with a diamond knife and stained with uranyl acetate and lead citrate. Sections were examined with TEM (JEM-1400FLASH, JEOL, Japan).

### Labeling of selected leaves with ^13^CO_2_

To explore the role of ILs and MLs in plant development under different light intensities, the ILs or MLs were labeled with ^13^CO_2_. After 20 d of light treatment, six pots (three for IL labeling and three for ML labeling) in each light treatment were subjected to ^13^CO_2_ labeling. Leaf labeling was performed 1 h after the start of illumination. Similar sizes of the ILs or MLs were carefully covered with a homemade sealing bag (sealed with foam glue). A syringe was used to inject 40 mL of ^13^CO_2_ gas into the bag, and 30 min later, the marking device was disassembled.

Two weeks after labeling, the plants (ILs labeled, MLs labeled, and unlabeled) were harvested and oven-dried at 70°C for 3 d for an assay of ^13^C abundance in the ILs, MLs, and RO. The movement of ^13^C under different light conditions was monitored; we used the labeled part as the denominator and the unlabeled part as the numerator, which reflects the amount transferred from labeled to unlabeled. A 0.2-mg plant sample was precisely weighed, and the ^13^C/^12^C ratio was measured using a stable isotope mass spectrometer (Delta V Advantage, Thermo Fisher Scientific, Germany).

### Determination of foliar P fractions and rhizome total P

The four fractions of foliar P (lipid P, nucleic acid P, metabolic P (including Pi), and residual P) were extracted from 50 mg of freeze-dried ILs and MLs according to Hidaka and Kitayama (2013) with modifications. The plant samples were digested with a mixed acid solution (nitric acid: perchlorate = 3:1, v/v) to extract different P fractions. The RO of total P was extracted by sulfuric acid. P was determined by the molybdenum blue method using a spectrophotometer (UV-6100, Mapada, Shanghai, China) (AMSs, 1966).

### Determination of alkaloid content using high-performance liquid chromatography

The determination of the alkaloid content of *C. chinensis* in RO was performed after 35 d of light intensity treatment according to the method of Qi Qi et al. (2018) with high-performance liquid chromatography (HPLC) (LC-20AT, Shimadzu, Japan). The lyophilized RO was weighed and ultrasonically dissolved in a hydrochloric acid–methanol solution (1:100, v/v). An Xtimate C18 chromatographic column (250 mm × 4.6 mm, 5 μm) (Welch, China) was applied to separate different alkaloid compounds, and the column temperature was 25°C. The mobile phase included acetonitrile (A) and 30 mmol/L ammonium bicarbonate solution (consisting of 0.7% ammonia and 0.25% triethylamine) (B) at a flow rate of 1 mL/min. The gradient program was set as follows: 0–15 min, 90%–75% B; 15–25 min, 75%–70% B; 25–50 min, 70%–55% B. The injection volume was set as 10 μL, and the detection wavelength was set at 270 nm.

### Statistical analysis

A one-way analysis of variance was performed to compare the data between the light-intensity experiments. The data was compared between two light intensity treatments or between the two sampling times of the same light intensity treatment using Tukey’s honestly significant difference test. All statistical analyses were performed using the software SPSS 25.0 (SPSS Inc., USA). The figures were drawn using Origin 2019 (Origin, USA).

## Results

### Chlorophyll *a* fluorescence in immature leaves and mature leaves

**Figure 1:**
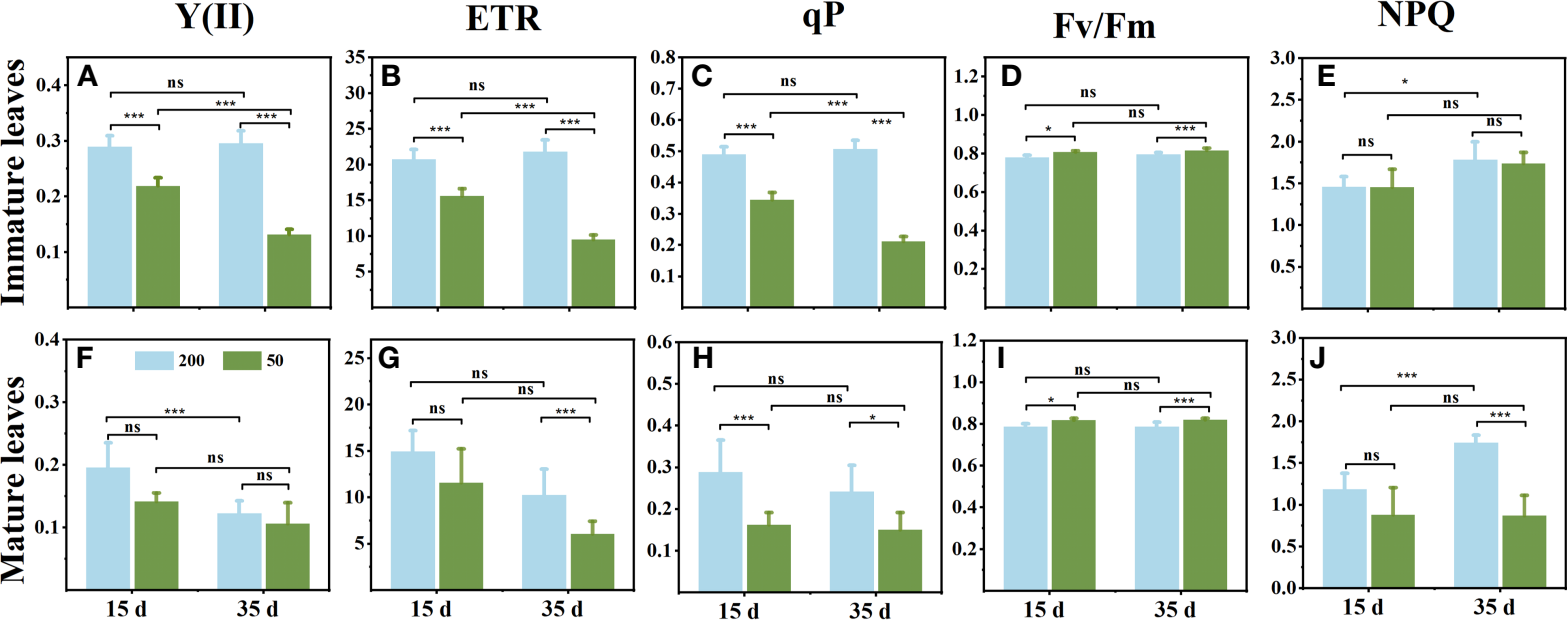
Changes of chlorophyll fluorescence parameters in immature leaves **(A–E)** and mature leaves **(F, G)** at 15 d and 35 d under 50 μmol m^−2^ s^−1^ and 200 μmol m^−2^ s^−1^ light intensity treatment. **(A, F)** The actual photochemical efficiency PSII [*Y*(II)]. **(B, G)** Relative electron transport rate (ETR). **(C, H)** Photochemical quenching (qP). **(D, I)** The maximum photochemical efficiency PSII (*F_v_/F_m_*). **(E, J)** Nonphotochemical dissipation of absorbed energy (NPQ). Each column represents the mean (± SE) of three replicates. Significant difference between the treatments: ^***^ *p* ≤ 0.001 (*t*-test); ^*^ *p* ≤ 0.05 (*t*-test); ns, no difference.

Light intensity affected the photochemistry of *C. chinensis* leaves during the leaf expansion stage. The *Y*(II), ETR, and qP in ILs under 200 μmol m^−2^ s^−1^ were significantly higher than those exposed to 50 μmol m^−2^ s^−1^ light intensity (**Figures 1A–C**). However, in MLs, the *Y*(II) showed no significant difference between the two light treatments (**Figure 1F**), and there was no difference between the observed ETR and qP between the two sampling times (**Figures 1G, H** **)**. Compared to relatively high light, the *F_v_/F_m_* in leaves was higher when the plants were under low light, but there was no change at the two sampling times (**Figures 1D, I** **)**. In ILs, light intensity had little effect on NPQ (**Figure 1E**), while NPQ in MLs under relatively high light was significantly higher than that under low light (**Figure 1J**).

### Chlorophyll concentration in immature leaves

**Figure 2:**
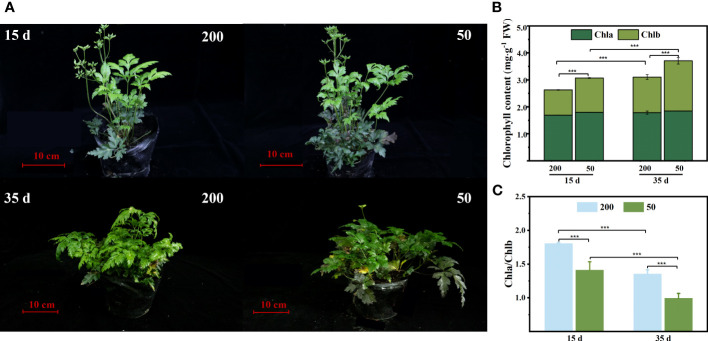
Chlorophyll concentration of immature leaves (IL) at 15 d and 35 d under 50 μmol m^−2^ s^−1^ and 200 μmol m^−2^ s^−1^ light intensity treatment. **(A)** Plant phenotypic. **(B)** Chlorophyll *a* (Chl *a*), chlorophyll *b* (Chl *b*), and total chlorophyll content (Chl *a* + Chl *b*). **(C)** The ratio of Chl *a* to Chl *b*. Each column represents the mean (± SE) of three replicates. Significant difference between the treatments: ^***^ *p* ≤ 0.001 (*t*-test). FW, fresh weight.

The plant phenotype observed that ILs turn green quickly under low light (**Figure 2A**). The chlorophyll concentration in ILs was consistent with our observations that decreasing light intensity increased the chlorophyll concentration, particularly Chl *b* concentration, and subsequently decreased the ratio of Chl *a/*Chl *b* ratio (**Figures 2B, C** **)**.

### Sucrose, glucose, and starch concentrations in leaves and rhizomes and starch morphology in immature leaves

**Figure 3:**
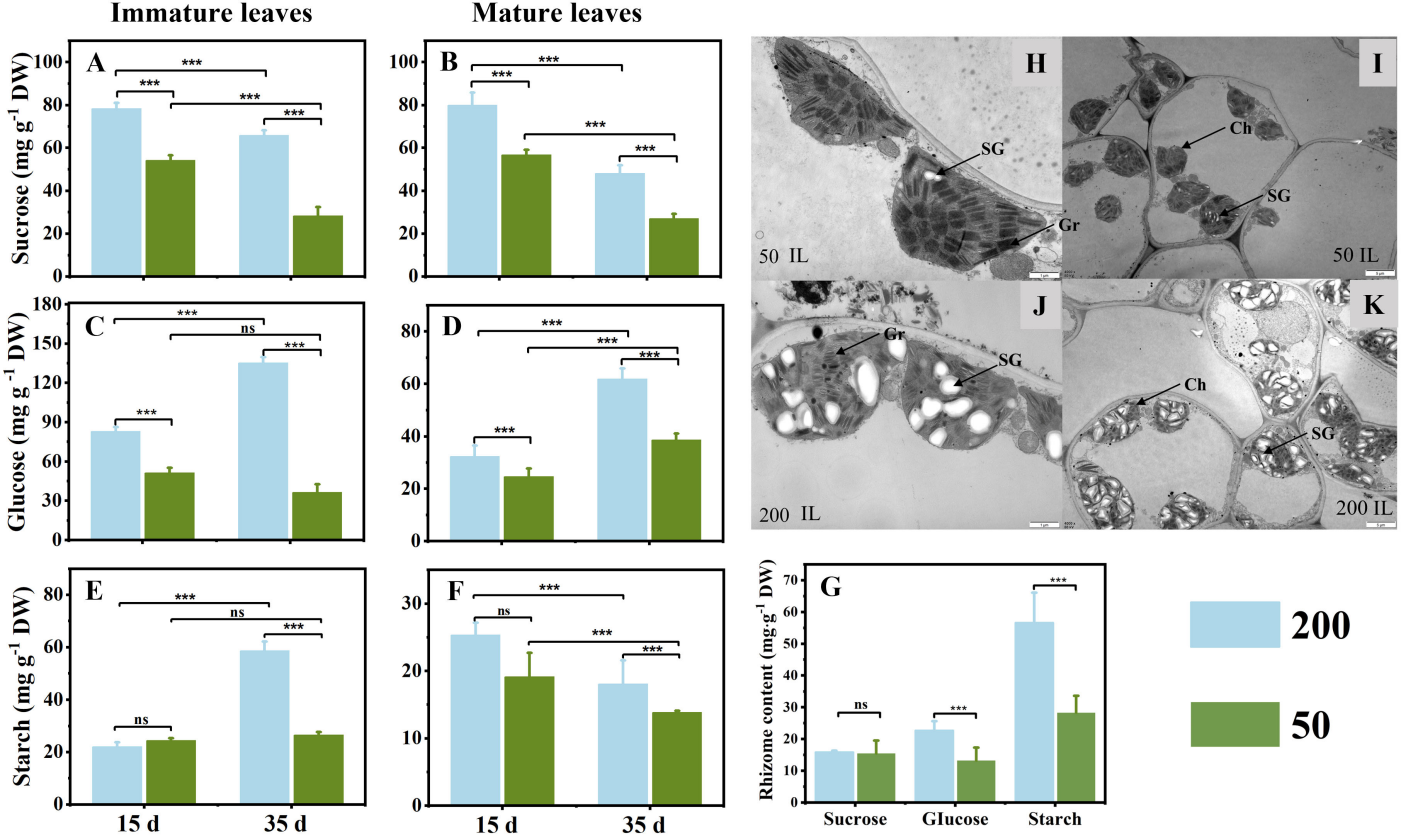
Sucrose **(A, B)**, glucose **(C, D)**, and starch **(D, E)** concentrations in immature leaves **(A, C, E)** and mature leaves **(B, D, F)** at 15 d and 35 d under 50 μmol m^−2^ s^−1^ and 200 μmol m^−2^ s^−1^ light intensity treatment. **(G)** Sucrose, glucose, and starch concentrations in rhizome at 35 d under two light intensities. **(H–K)** The ultrastructures of chloroplasts in ILs after 35 d of light treatment were observed under a transmission electron microscope (TEM); **(H, J)** ×15,000, **(I, K)** ×4,000. Ch, chloroplast; Gr, grana; SG, starch grain. Each column represents the mean (± SE) of three replicates. Significant difference between the treatments: ^***^ *p* ≤ 0.001 (*t*-test); ns, no difference between the treatments. DW, dry weight.

Light intensity affected the sucrose, glucose, and starch concentrations in ILs, MLs, and RO at the two sampling times (**Figure 3**). The sucrose concentration in ILs and MLs under 200 μmol m^−2^ s^−1^ light intensity was higher than that under 50 μmol m^−2^ s^−1^ light intensity, and it was higher at 15 d compared to 35 d (**Figures 3A, B** **)**. Compared with plants under low light intensity, relatively high light intensity increased the glucose concentration at both sampling times, irrespective of the age of the leaves (**Figures 3C, D** **)**. In ILs, the starch concentration showed no difference between the two light treatments at 15 d, but in relatively high light, it was significantly higher than that in the low light at 35 days (**Figures 3E, F** **)**. In MLs, the starch concentration showed a decrease from 15d to 35 d under both two light treatments, which was decreased further under low light (**Figure 3F**). After 35 d light intensity treatment, the starch and glucose in the RO under 200 μmol m^−2^ s^−1^ were higher than those under 50 μmol m^−2^ s^−1^ (**Figure 3G**). In addition, the results of the electron microscope photography were consistent with the results obtained above. The chloroplasts were full of starch granules under 200 μmol m^−2^ s^−1^, but almost no starch granules were observed under 50 μmol m^−2^ s^−1^ (**Figures 3H–K**).

### The atomic percentage of ^13^C in different plant parts

**Figure 4:**
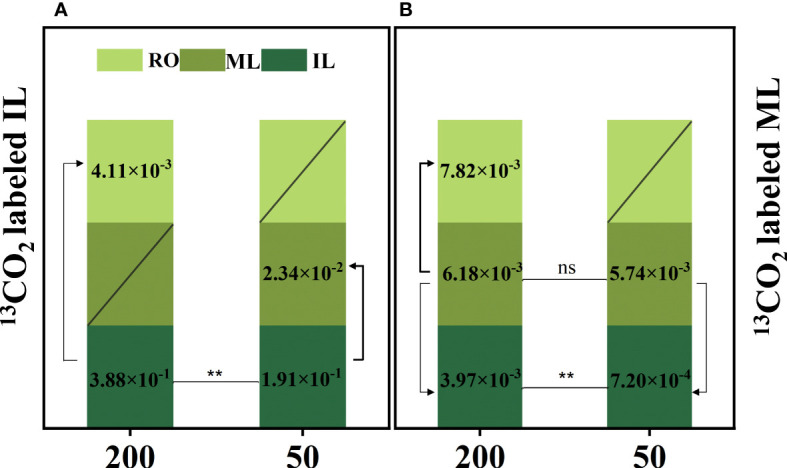
The atomic percentage of ^13^C [ AT%=C 13/(C 13+C 12)×100% ] in immature leaves (IL), mature leaves (ML), and rhizomes (RO) after ^13^C-labeled IL **(A)** and ML **(B)**. Data represent the atomic percent of ^13^C, “/” indicates the ^13^C atom was not detected. Each column represents the mean (± SE) of three replicates. Significant difference between the treatments: ^**^ *p* ≤ 0.01 (*t*-test); ns, no difference between the treatments. The thickness of the line represents the amount of transfer.

^13^C labeling plays an important role in monitoring carbon flow. The proportion of ^13^C in ILs under 200 μmol m^−2^ s^−1^ was significantly higher than that under 50 μmol m^−2^ s^−1^, regardless of whether ILs or MLs were labeled with ^13^C. Furthermore, under 200 μmol m^−2^ s^−1^ treatment, ^13^C atoms were detected in ILs and RO but not in MLs when ^13^C was labeled in ILs. Under 50 μmol m^−2^ s^−1^ light, the ratio of mature-to-immature leaf ^13^C concentration in the treatment of IL ^13^C labeling (12.2%) was higher than the ratio of immature-to-mature leaf ^13^C concentration in the treatment of ML ^13^C labeling (7.3%), ^13^C atoms were detected in ILs and MLs but were not in the RO (**Figure 4**).

### Concentrations of total P in leaves and rhizomes and foliar P fractions

**Figure 5:**
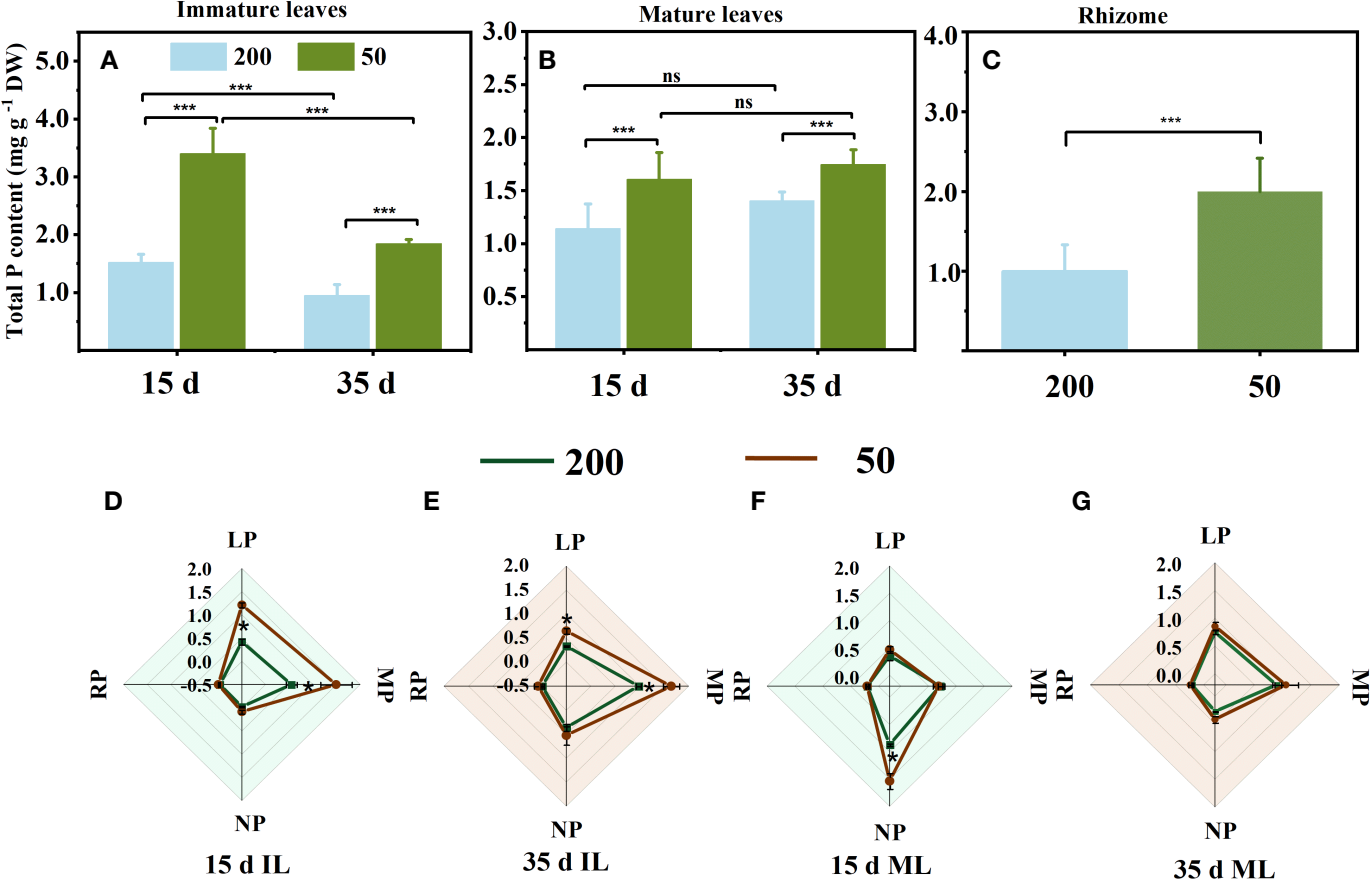
Total phosphorus concentration in immature leaves (IL) **(A)**, mature leaves (ML) **(B)**, and rhizome **(C)**, and foliar phosphorus fractions concentration **(D–G)** at 15 d and 35 d under 50 μmol m^−2^ s^−1^ and 200 μmol m^−2^ s^−1^ light intensity. Each column represents the mean (± SE) of three replicates. Significant differences between the treatments: ^***^ *p* ≤ 0.001 (*t*-test); ^*^ *p* ≤ 0.05 (*t*-test); ns, no difference between the treatments. DW, dry weight; RP, residual P; LP, lipid P; MP, metabolite P; NP, nucleic P.

The total P concentration in leaves and RO under 200 μmol m^−2^ s^−1^ was lower than that under 50 μmol m^−2^ s^−1^ (**Figures 5A–C**). Under 50 μmol m^−2^ s^−1^ light intensity, the leaf P concentration of MLs showed no difference observed at both sampling times (**Figure 5B**). However, the leaf P concentration of ILs under 200 μmol m^−2^ s^−1^ light intensity was lower than that under 50 μmol m^−2^ s^−1^ light intensity at both sampling times (**Figure 5A**). Light not only affected the foliar total P concentration but also the foliar P fractions, especially the organic P (**Figures 5D–G**). In ILs, the lipid P and metabolite P under relatively high light were significantly lower than those under low light intensity at both sampling times (**Figures 5D, E** **)**. At 15 d, the main sources of phosphorus in ILs were lipid P and metabolic P (**Figure 5D**), but metabolic P and nucleic P had a high proportion at 35 d (**Figure 5E**). In MLs, only nucleic P increased with decreasing light intensity at 15 d (**Figure 5F**). Nucleic P had a high proportion in MLs at 15 d, and the proportion of lipid P and metabolic P increased at 35 d compared to 15 d (**Figures 5F, G** **)**. Furthermore, the residual P showed no difference in ILs and MLs under the two light intensity treatments at both measuring times (**Figures 5D–G**).

### The concentration of alkaloids in the rhizome and biomass accumulation, and the correlation between alkaloids and starch in the rhizomes

**Figure 6:**
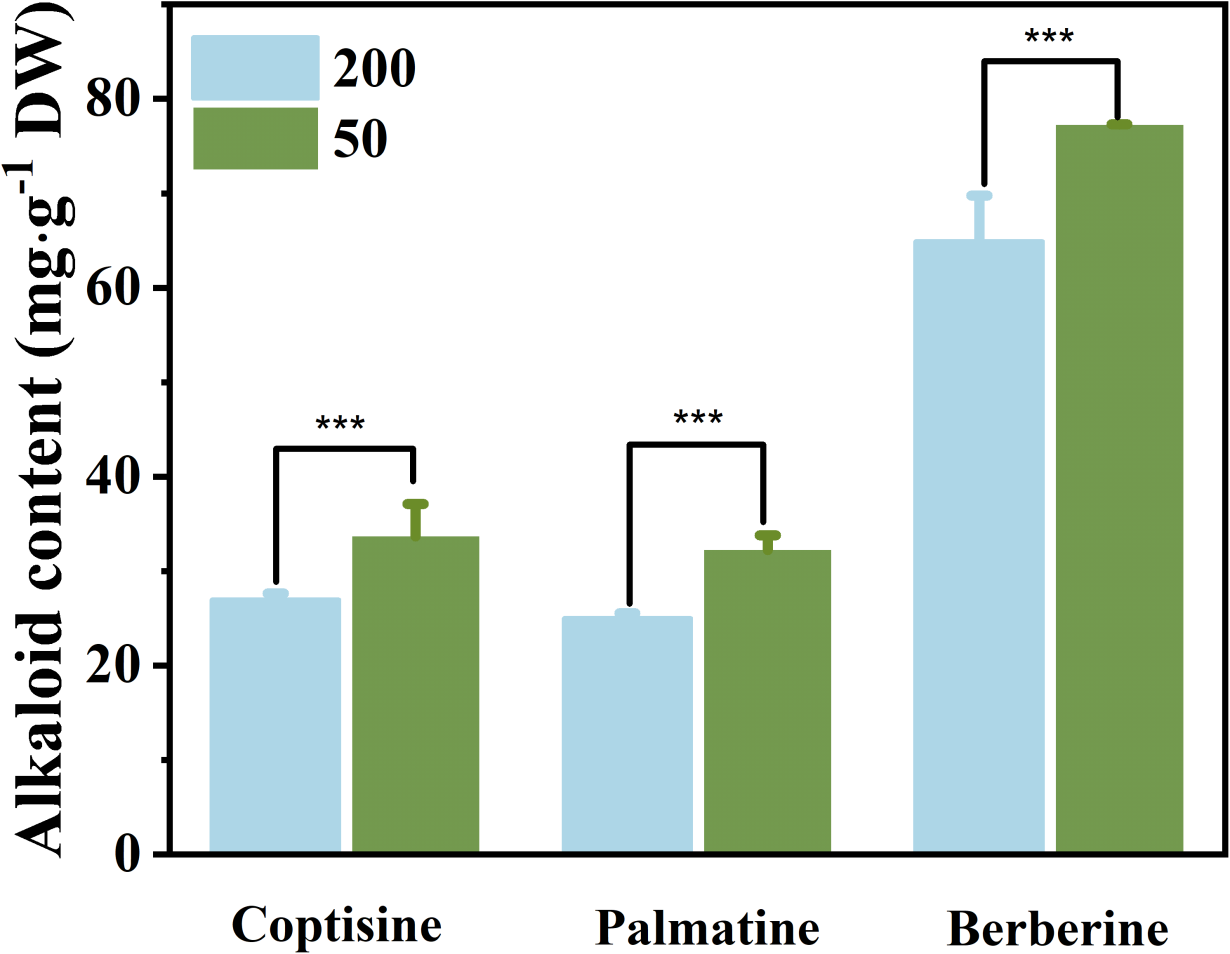
The concentration of three kinds of alkaloids in rhizomes under 50 μmol m^−2^ s^−1^ and 200 μmol m^−2^ s^−1^ light treatments after 35 d. Each column represents the mean (± SE) of three replicates. Significant difference: ^***^ *p* ≤ 0.05. DW, dry weight.

The findings demonstrated that under low light conditions, the alkaloid concentrations in the RO of berberine (23.0%), coptisine (19.9%), and palmatine (17.9%) were all greater than those under relatively high light intensity (**Figure 6**). The light treatment at the leaf expansion stage has an effect on the biomass of RO at the mature stage. The RO dry weight of plants under relatively high light at the mature stage was significantly higher than that under low light (**Supplementary Figure S6**).

**Figure 7:**
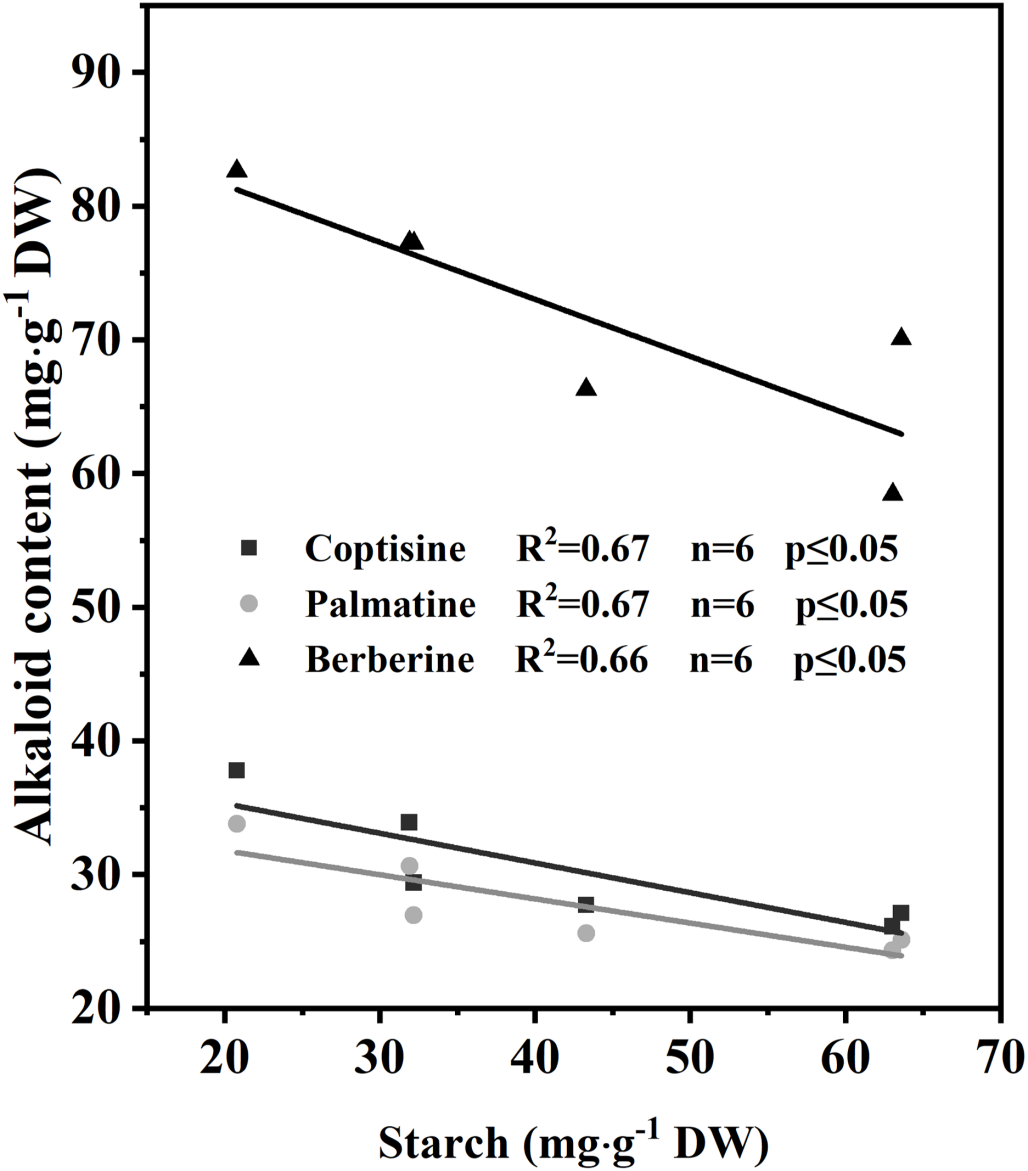
Correlation of alkaloids with starch concentration in rhizomes after 35 d of light treatment (*n* = 6).

The relationships between the starch concentration in RO and the concentration of the three main alkaloids in RO were investigated *via* regression analyses (**Figure 7**). The concentrations of the three main alkaloids (**Figure 7**) were negatively correlated with starch concentration in RO (*n* = 6; *R* ^2 = ^0.67, 0.67, 0.66; *p* ≤ 0.05), indicating that the lower the starch concentration, the higher the alkaloid concentrations.

## Discussion

### Light intensity changed the sink–source relationships among ILs, MLs, and RO during the expansion of ILs

The initial growth stages of ILs primarily rely on imported reserves from source organs such as MLs, stems, and RO, but eventually become net exporters of carbon and rely on their own photosynthates (Miyazawa et al., 2003). High light intensity enhances local photosynthesis, which induces the transfer of produced carbohydrates to source organs, reducing the consumption of reserves in source organs to supply new leaf growth, making reserves more durable, and ultimately increasing their biomass (Rossi et al., 2015; Feng et al., 2019; Wimalasekera, 2019; Zhou et al., 2022). Our findings confirm that increased carbon assimilation by ILs and MLs under high light during leaf expansion reduces RO reserve consumption (**Figure 4**), ultimately increasing RO biomass (**Supplementary Figure S6**). Additionally, high light accelerates the conversion of ILs from sink to source, which reduces RO reserve consumption. However, we observed that MLs exacerbated the depletion of RO reserves as a sink under low-light conditions, which contrasts with prior studies.

In *C. chinensis*, a delayed-greening species, leaves from emergence to maturity take at least 45–60 d. In this process of leaf maturation, ILs, MLs, and RO are involved in a high intensity of reserve exchange. In most dicotyledonous plants, the leaf changes from sink to source when the leaf area reaches 10%–60% of the final area (Miyazawa et al., 2003; Pantin et al., 2012). In the present study, some ILs were not fully expanded 15 d after treatment, and all had reached the maximum area by 35 d, but the chlorophyll concentration in ILs was lower than MLs (**Figure 2**). Furthermore, the *F_v_/F_m_* showed no difference between the two sampling times and reached a high level above 0.8 (**Figures 1D, I** **)**. The amount of glucose and starch, the major carbohydrate forms for transport and storage, increased markedly from 15 d to 35 d in ILs (**Figures 3C, E** **)**. These results are consistent with the typical characteristics of the transition from the sink to the source of ILs in the studies that have been reported (Turgeon, 1989; Czech et al., 2009). Indeed, the growth of ILs in *C*. *chinensis* at least partially depends on its photosynthesis from 15 d to 35 d after emergence of leaves. The glucose and starch concentrations in ILs increased markedly from 15 d to 35 d when plants were grown under 200 μmol m^−2^ s^−1^ (**Figures 3C, E** **)**. In contrast, under 50 μmol m^−2^ s^−1^, the glucose and starch concentrations in ILs remained at very low levels and did not show significant changes at the two sampling times (**Figures 3C, E** **)**. Interestingly, the glucose concentration in the RO was lower in plants grown under 200 μmol m^−2^ s^−1^ than those grown under 50 μmol m^−2^ s^−1^ at 35 d (**Figure 3G**), suggesting that starch breakdown was triggered to support shoot growth under low light conditions (Hajirezaei et al., 2003; Stitt and Zeeman, 2012). Additionally, ^13^C was detected in the RO of the ILs ^13^C label under 200 μmol m^−2^ s^−1^ but not under 50 μmol m^−2^ s^−1^ (**Figure 4**). These findings indicate that increasing light intensity before leaf maturation in *C. chinensis* can significantly enhance the autotrophic capacity of ILs by providing carbohydrates to support their own growth and transporting carbohydrates underground to accumulate RO biomass. In contrast, under low light, the RO continues to supply the growth of ILs, leading to increased consumption of reserves in the RO.

The MLs of evergreen species can resume their functions in early spring to sustain themselves and provide partial resources to support the new leaf growth as the temperature rises (Walters and Reich, 1999; Ida and Kudo, 2008). Our findings reveal that the functional performance of MLs is mainly influenced by the light intensity to which they are exposed. Under high light intensity, a higher proportion of ^13^C was detected in the RO and ILs when MLs were labeled with ^13^C. This observation provided evidence to support the view that MLs provide partial resource support for IL growth (Turgeon, 1989). No difference in starch concentration was observed in the MLs between the two light intensity treatments at 15 d (**Figure 3F**). However, at 35 d, MLs under low light exhibited a lower starch concentration compared to those under high light (**Figure 3F**). These suggest that under 50 μmol m^−2^ s^−1^, MLs rely on stored starch to ensure their survival (Stitt & Zeeman, 2012). Furthermore, the ratio of ^13^C in mature-to-immature leaf in IL ^13^C labeling was 12.2%, and the ratio of ^13^C in immature-to-mature leaf in ML ^13^C labeling was 7.3% under 50 μmol m^−2^ s^−1^ light intensity (**Figure 4**). Moreover, under 200 μmol m^−2^ s^−1^, the ^13^C was not even detected in MLs in IL ^13^C labeling (**Figure 4**). Therefore, under low light, MLs did not have additional carbon to sustain IL growth, but instead received energy from the ILs.

Shade-tolerant plants have evolved a trade-off between growth potential and survival in low light, such as increasing their photosynthesis efficiency and decreasing the respiration rate to decrease the consumption of energy when the plants are grown in deep shade (Loach, 1967; Reich et al., 1998; Yao et al., 2017). However, in our study, MLs maintained the same respiration rate under low light intensity as under high light and remained at a high level (data not shown), which is inconsistent with previous observations in other species (Loach, 1967; Reich et al., 1998; Yao et al., 2017). One possible explanation for this discrepancy might be that the 50-μmol m^−2^ s^−1^ light intensity harmed the carbon fixation of *C. chinensis* but did not cause severe stress (**Supplementary Figure S5**). For example, the *F_v_/F_m_*, a sensitive indicator of plant responses to environmental stresses (Baker, 2008), showed no difference between 15 d and 35 d in the two samples, regardless of leaf age (**Figures 1D, I** **)**. Additionally, *C. chinensis* is typically found in mountainous regions at elevations of 1,000–2,000 m. During the new leaf growth period (from February to April), the average day/night temperature is around 15°C/5°C (**Supplementary Figure S2D**). Under low temperatures, *C. chinensis*, like other shade-tolerant plants, can survive under low light by reducing their respiration rate (Walters and Reich, 1999; Walters and Reich, 2000). However, the day/night temperatures set in this experiment were higher than those found in its natural habitat. To ensure survival, MLs may need to utilize energy from other organs. Nevertheless, the strategy of MLs of shade-tolerant perennial plants to adapt to low light stress at higher temperatures by consuming energy provided by other organs to sustain survival rather than rapid senescence needs more evidence.

The energy consumption in the source organ (especially RO) by flowering was not considered in this experiment. This may have potentially amplified the result indicating that light-mediated IL growth increases the consumption of reserves in the source organ (RO), particularly under low-light conditions. However, the amount of reserves in the RO did not change the source–sink relationship among ILs, MLs, and RO, which is mainly determined by the light-regulated carbon assimilation capacity of ILs and MLs.

### Relatively high light decreased the P demand needed to maintain the photosynthesis potential of immature leaves

The expansion of new leaves in understory plants during spring is mainly supported by stored P rather than uptake by roots in the current season (Schachtman et al., 1998; Rychter and Rao, 2005). The low P concentration in the new leaves suggests a low total of P export from the source organ. Our data are consistent with this observation, as the ILs of plants grown under 200 μmol m^−2^ s^−1^ had lower P concentration than those grown under 50 μmol m^−2^ s^−1^ (**Figure 5A**). In addition, the *Y*(II) *of* ILs under 200 μmol m^−2^ s^−1^ was higher than those under 50 μmol m^−2^ s^−1^ (**Figure 1A**).

Relatively high light increased photosynthesis efficiency in ILs associated with lower P concentrations in two ways. First, the level of enzymes involved in Rubisco and other Calvin–Benson cycles is mainly connected to the amount of rRNA, which is mainly determined by the amount of nucleic P (Rychter and Rao, 2005; Hussain et al., 2021). The high light intensity did not decrease the nucleic P concentration in the ILs compared to that under low light (**Figures 5D, F** **)**. Second, under high light intensity, a lower number of chloroplasts and a low chlorophyll concentration will decrease the investment of P in protein synthesis and cellular growth (Elser et al., 2010; Veneklaas et al., 2012). Light intensity rather than chlorophyll concentration is the main factor limiting photosynthesis (Rychter and Rao, 2005; Hussain et al., 2021). Our data are consistent with this observation, as the chloroplast number and chlorophyll concentration in ILs of plants grown under 200 μmol m^−2^ s^−1^ were lower than those of ILs grown under 50 μmol m^−2^ s^−1^ (**Figures 2B**, **3H–K**). Furthermore, when the plants were grown under 50 μmol m^−2^ s^−1^ light intensity, the increased chlorophyll concentration was mostly attributed to a high proportion of Chl *b* (**Figures 2B, C** **)**, which is currently believed to improve light harvesting under low light conditions and has been demonstrated in numerous earlier investigations (Escoubas et al., 1995; Sun et al., 2003; Valladares and Niinemets, 2008; Melgar et al., 2009; Feng et al., 2019).

**Figure 8:**
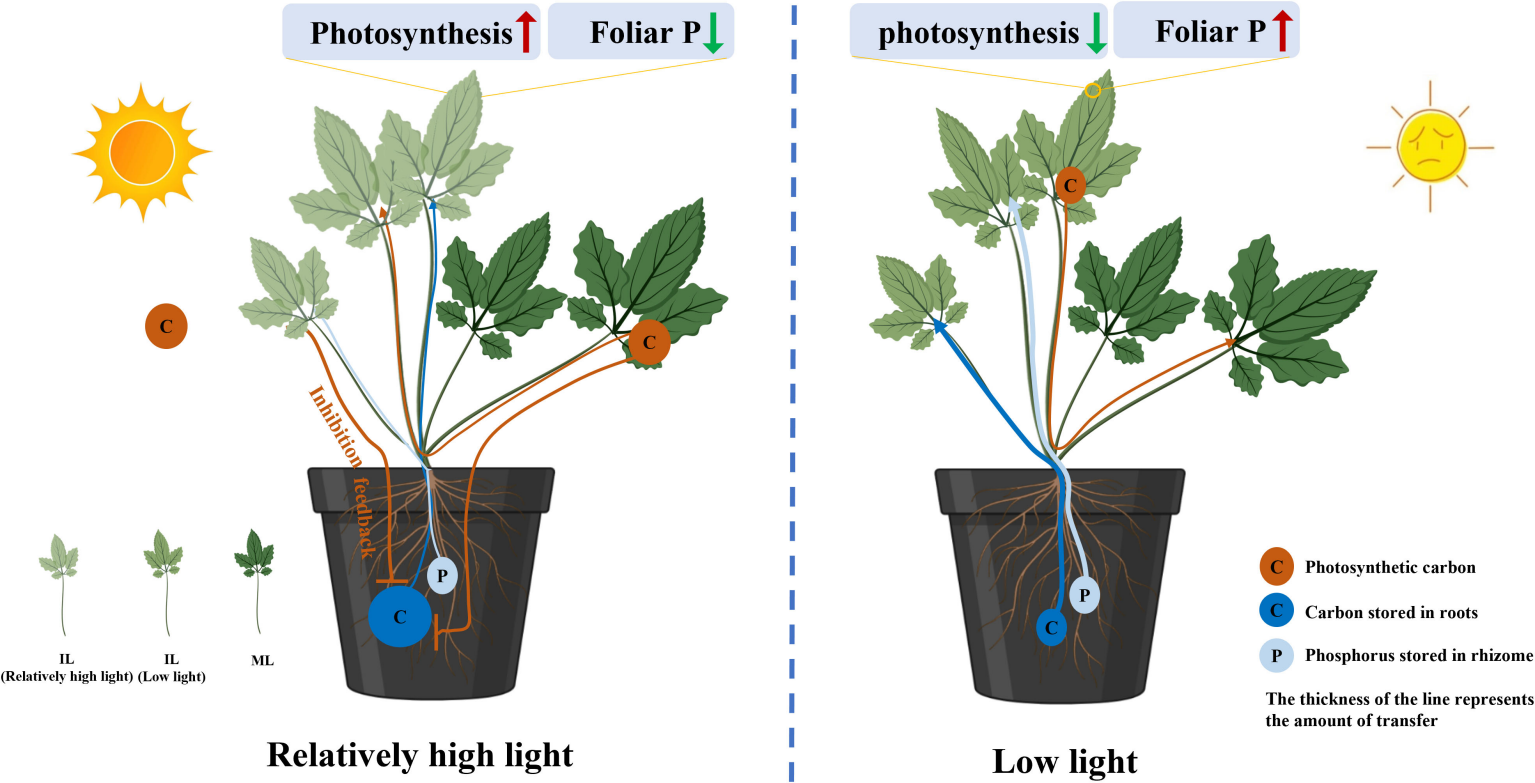
Schematic of a proposed model showing the decreased consumption of reserves in the rhizomes (RO) relating to the source–sink relationship changes regulated by light intensity. Relatively high light intensity during the period of leaf expansion increased the photosynthetic capability of immature leaves (ILs) that are associated with low P concentration. The increased carbon capture and decreased P demand in the ILs inhibited the transport of carbohydrates and P from the RO to the leaves. In addition, under low light, barely any reserves are exported from mature leaves (MLs) to feed ILs growing, which aggravates the amount of reserves lost in the RO. The thickness of the line represents the amount of transfer.

The P concentration in the RO of *C. chinensis* grown under 200 μmol m^−2^ s^−1^ was lower than that of plants grown under 50 μmol m^−2^ s^−1^, which may challenge the view that high light suppresses the amount of P exported from the RO. One possible explanation is due to the concentration effect (**Supplementary Figure S6**) (Jarrell and Beverly, 1981). Under 50 μmol m^−2^ s^−1^, the amount of carbohydrates was much higher than P from RO to supply the growth of ILs, resulting in an increased P concentration in the RO. Indeed, relatively high light intensity reduced the demand for P to maintain the photosynthesis potential of ILs, thus inhibiting the export of P from the RO (**Figure 8**).

### Effects of light on the rhizome alkaloid concentration

Rather than directly mediating synthesis, low light promotes the increase in alkaloid concentration in *C. chinensis* RO in early spring by mediating substance translocation. However, low light significantly reduces RO biomass (**Supplementary Figure S6**). In most evergreen plants, nonstructural carbohydrate concentrations typically reach their maximum in the fall, decline during the winter, and reach minimum levels immediately after growth initiation in the spring (Wyka, 1999). The alkaloid concentration in the RO of *C. chinensis* showed two peaks in March (new leaf expansion) and September (vigorous growth period), with the concentration in September being significantly higher than that in March (Ke, unpublished; Zhao and Du, 2002). RO alkaloids and nonstructural carbohydrates were negatively correlated in March and positively correlated in September. Secondary metabolism usually uses the end products of primary metabolism as substrates (Drew and Demain, 1977), suggesting that the increase in RO alkaloids in March may not be regulated by synthesis.

In the present study, RO under low light in the early spring had lower starch and higher alkaloid concentration than that of plants under high light (**Figure 6**). Furthermore, there was a negative correlation between starch and alkaloid concentration (**Figure 7**). The increase in alkaloid concentration mainly attributed to the concentration effect (Jarrell and Beverly, 1981), as the amount of carbohydrates in the RO supplying ILs growth was higher under low light than under high light, resulting in an increased alkaloid concentration in the RO. These findings support our view, but further exploration is needed to understand the effect of light on alkaloid synthesis in *C. chinensis*.

## Conclusions

Our findings have demonstrated that during leaf expansion, the RO of *C. chinensis* had lower carbohydrate and P losses under 200 μmol m^−2^ s^−1^ compared to plants under 50 μmol m^−2^ s^−1^. After rapid leaf expansion, the high light intensity can enhance the photosynthetic capacity and sink to the source transition rate of ILs, thereby reducing reserve consumption in RO. Under high light, MLs served as a source to provide carbohydrates for IL growth, reducing the consumption of reserves in RO. Conversely, under low light, MLs have low photosynthesis efficiency but maintain relatively high metabolic activity, acting as a sink to survive and supported by other organs, which aggravates the reserves lost in the RO. Furthermore, under high light intensity, less P is required in ILs to maintain a high rate of photosynthesis, resulting in less P distributed from RO to ILs (**Figure 8**). Nevertheless, the regulation of light on the synthesis and accumulation of alkaloids in RO requires further investigation. This study found that light accelerates the development of photosynthetic capacity in ILs, resulting in reduced reserve consumption in the RO and ultimately leading to increased yield. Under low light and high-temperature conditions, MLs of *C. chinensis* may survive by consuming energy supplied by other organs, which may be a new mechanism for its adaptation to environmental stress, but more evidence is needed. Nevertheless, it is recommended that light intensity be appropriately increased during the development of ILs to promote leaf development and reduce the reserve consumption in the source organ. However, further research is needed to determine the optimal intensity and duration of high-light exposure.

## Data availability statement

The original contributions presented in the study are included in the article/**Supplementary Material**. Further inquiries can be directed to the corresponding authors.

## Author contributions

TZ, WK, YM, and BX carried out the design of this research work and wrote this paper. WK, YL, FZ, and JD carried out the plant cultivation, chemical analysis, and statistical analysis of this work, and MP participated in experiment management and manuscript revision.
