# Supplementary material for: Relatively high light inhibits reserves degradation in the Coptis chinensis rhizome during the leaf expansion by changing the source-sink relationship
Source: Front Plant Sci. 2023 Sep 1;14:1225895. doi: 10.3389/fpls.2023.1225895 (PMC10502731; doi:10.3389/fpls.2023.1225895)
Supplement: Supplementary file 1 [file DataSheet_1.docx]

**
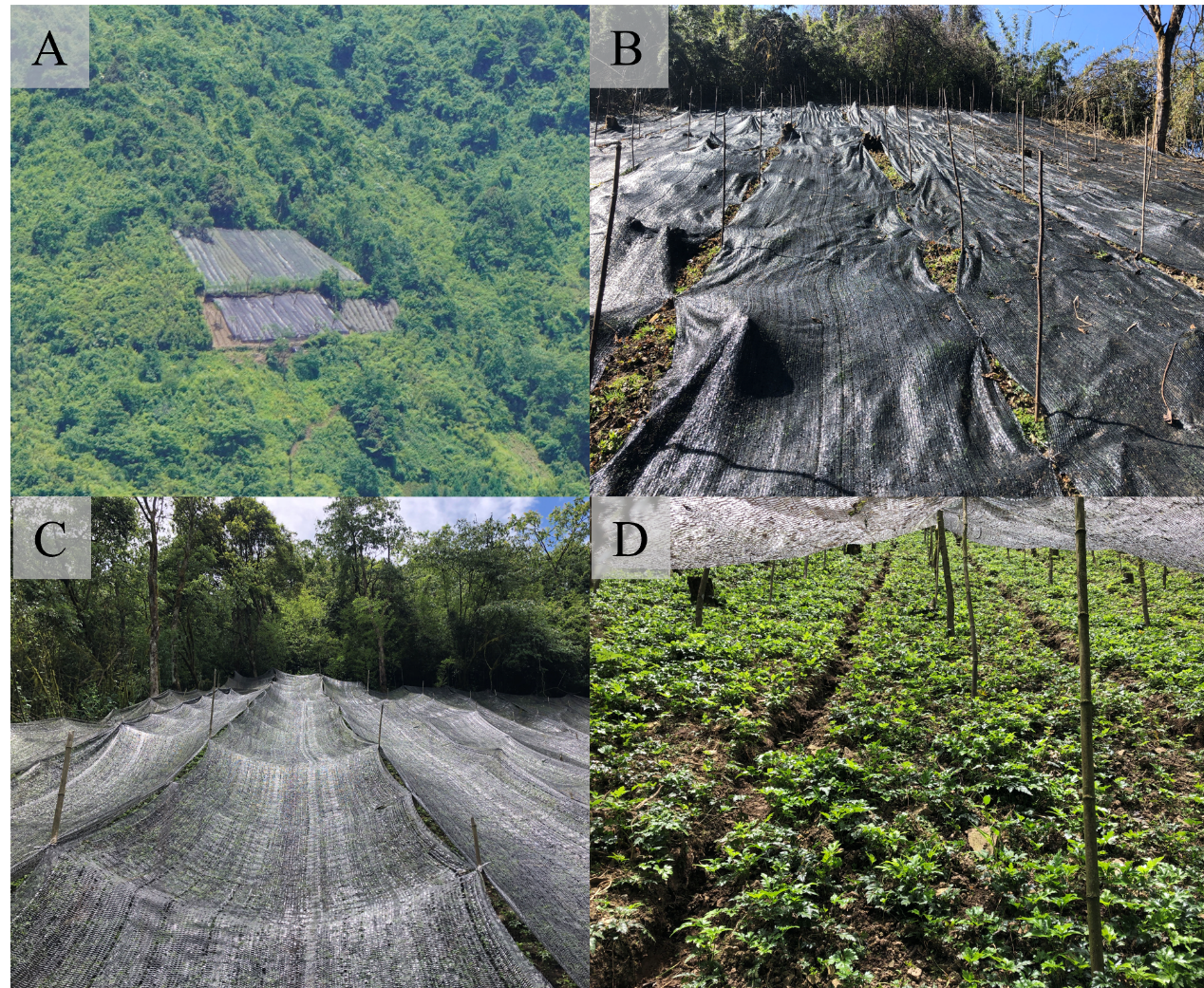
**

**Figure S1** The planting base of *Coptis chinensis*. A: artificial cultivation of *C. chinensis* after cutting trees; B: artificial shade of *C. chinensis* in winter (preventing snow from overpowering *C. chinensis*); C: artificial shade of *C. chinensis* in other seasons**;** D: the growth of *C. chinensis* under the shade.

**
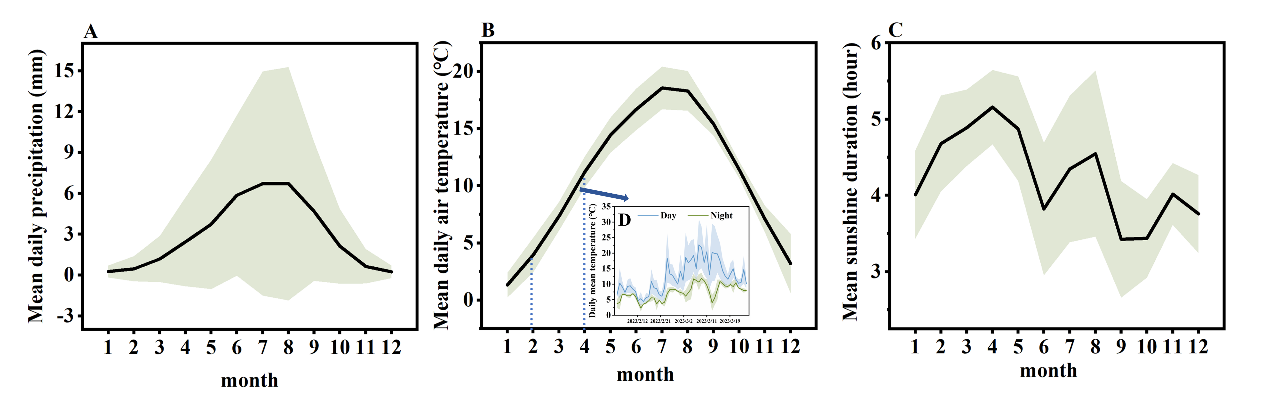
Figure S2** The average daily precipitation (A), average daily temperature (B), and average daily light duration (C) in the *Coptis* *chinensis* (Pengzhou, China) planting bas recorded over the past 20 years (from 2000 to 2020). The average daily day and night temperatures between February and April (D).

**
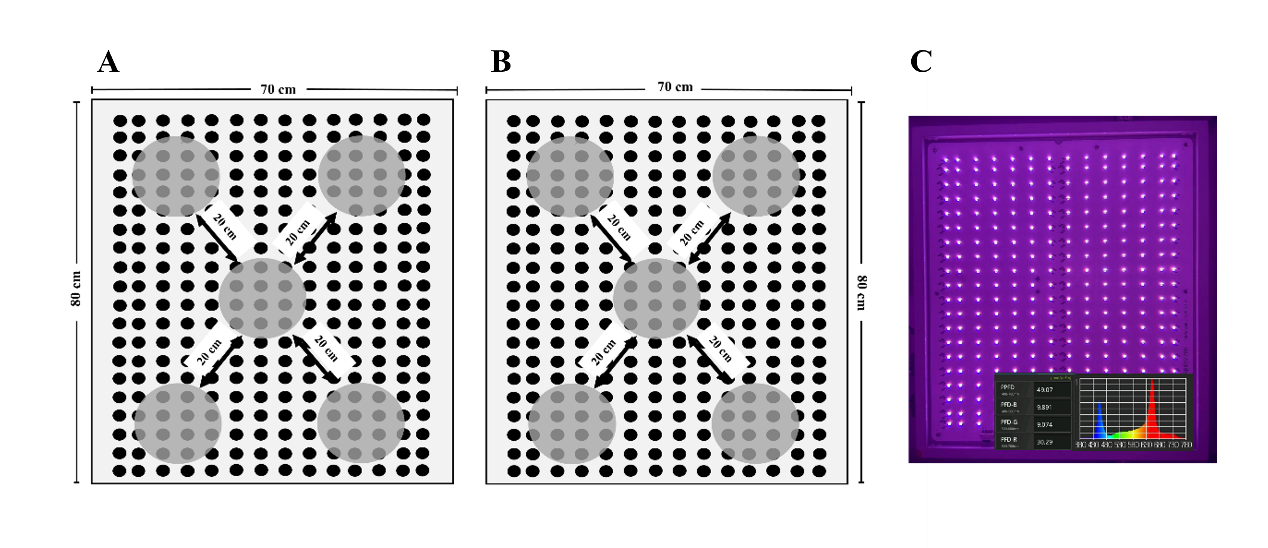
**

**Figure S3** Schematic of an arrangement of plants under the light-emitting diode (LED) lamp panel and lamp bead pattern on the panel. The large grey circle represents the pot; the black dots represent the lamp bead. A, plants under optimal light intensity. B, plant under low light intensity. C, photograph of the panel and spectral information of the lamps.


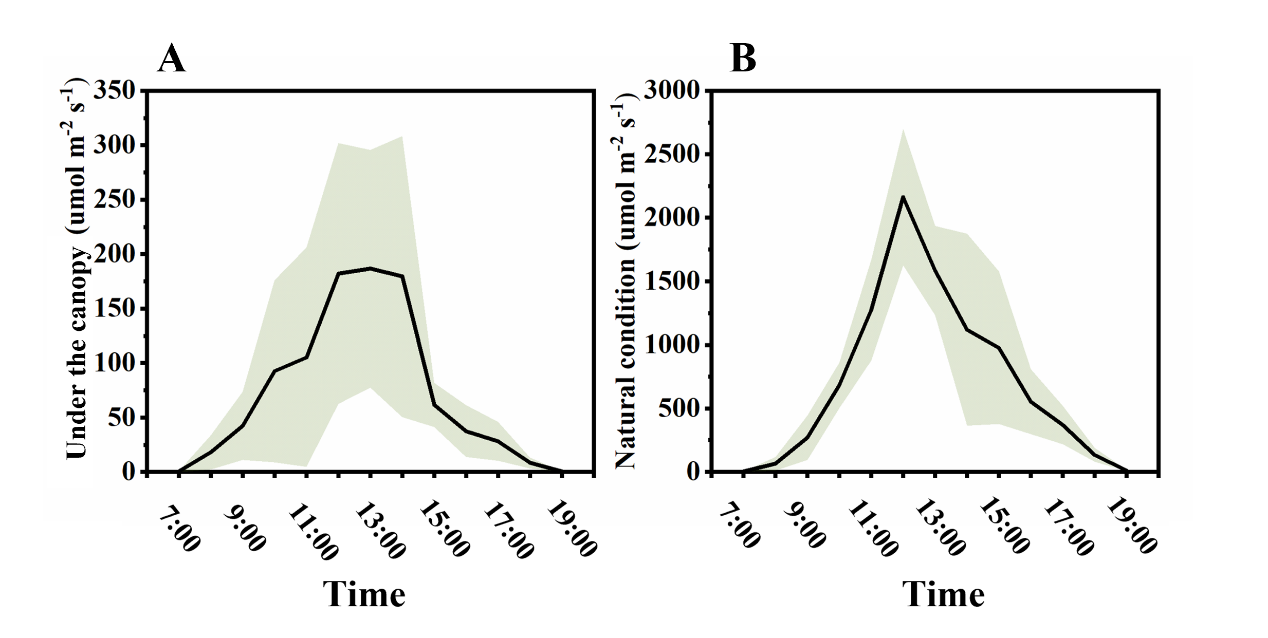


**Figure S4** Daytime light intensity under different conditions in early spring. A: Light intensity under the canopy; B: Light intensity under natural conditions.





**Figure S5** Single leaf area (cm^2^) after 35 d of treatment under different light intensities.

**

**

**Figure S6** Rhizome dry weight (DW) per plant harvested after one year.
