# Supplementary material for: Relatively high light inhibits reserves degradation in the Coptis chinensis rhizome during the leaf expansion by changing the source-sink relationship
Source: Front Plant Sci. 2023 Sep 1;14:1225895. doi: 10.3389/fpls.2023.1225895 (PMC10502731; doi:10.3389/fpls.2023.1225895)
Supplement: Supplementary file 2 [file Table_1.docx]

| **PAR** | 0 | 36 | 64 | 93 | 129 | 179 | 273 | 409 | 606 | 896 | 1174 |
| --- | --- | --- | --- | --- | --- | --- | --- | --- | --- | --- | --- |
| **ETR** | 0 | 6.7 | 8.7 | 9.7 | 10.1 | 10.1 | 10 | 9.6 | 8.1 | 7.9 | 7.4 |
|  | 0 | 5.1 | 6.7 | 8.7 | 9.9 | 10.2 | 10.0 | 9.5 | 7.8 | 7.7 | 6.5 |
|  | 0 | 6.6 | 7.6 | 8.9 | 10.3 | 10.5 | 10.4 | 9.3 | 8.6 | 7.4 | 6.9 |

**Table S1** Fast light response curve parameters of *Coptis* cultivated

**Table S2** Fast light response curve parameters of *Coptis* cultivated in two light intensities after 35 days

|  | **PAR** | 0 | 36 | 64 | 93 | 129 | 179 | 273 | 409 | 606 | 896 | 1174 |
| --- | --- | --- | --- | --- | --- | --- | --- | --- | --- | --- | --- | --- |
| **ETR** | **200 ML** | 0 | 4.5 | 6.9 | 8.1 | 8.9 | 9.4 | 9 | 8.9 | 8.8 | 8.8 | 7.2 |
|  | **200 IL** | 0 | 4.6 | 7.6 | 10.4 | 12.9 | 15.2 | 18.8 | 18.4 | 18.3 | 18.2 | 19 |
|  | **50 ML** | 0 | 2.9 | 2.6 | 3.4 | 4.1 | 4.8 | 5.4 | 5.9 | 5.6 | 5.8 | 7.1 |
|  | **50 IL** | 0 | 5.5 | 8.1 | 9.7 | 11.1 | 12.4 | 12.8 | 12 | 12.7 | 10.8 | 11.8 |
